# The LIVER CARE trial — screening for liver disease in individuals attending treatment for alcohol use disorder: a randomized controlled feasibility trial

**DOI:** 10.1186/s40814-024-01504-5

**Published:** 2024-05-16

**Authors:** Peter Jepsen, Natasja von Wowern, Lone Galmstrup Madsen, Mette Kruse Klausen, Signe Düring, Kirstine Skov Benthien, Matilde Winther-Jensen, Janne Petersen, Gro Askgaard

**Affiliations:** 1 Department of Hepatology and Gastroenterology, Aarhus University Hospital, Aarhus, Denmark; 2 Department of Medicine, Section of Gastroenterology and Hepatology, Zealand University Hospital, Køge, Denmark; 3 Institute for Clinical Medicine, University of Copenhagen, Copenhagen, Denmark; 4 Novavi Alcohol Treatment Center Køge, Køge, Denmark; 5 Psychiatric Center Copenhagen, Frederiksberg University Hospital, Copenhagen, Denmark; 6 Palliative Care Unit, Copenhagen University Hospital – Hvidovre, Hvidovre, Denmark; 7 Department of Data, Biostatistics and Pharmacoepidemiology, Center for Clinical Research and Prevention, Frederiksberg University Hospital, Copenhagen, Denmark; 8 Department of Public Health, Section of Biostatistics, University of Copenhagen, Copenhagen, Denmark

**Keywords:** Feasibility study, Alcohol use disorder, Screening, Alcohol-related liver disease, Outpatient alcohol treatment, Randomized trial

## Abstract

**Background:**

Alcohol-related liver disease is a preventable disease with high mortality. If individuals with alcohol-related liver disease were to be diagnosed earlier by screening and they reduced their alcohol consumption, lives lost to alcohol-related liver disease might be saved. A liver stiffness measurement (FibroScan©) is a key tool to screen for alcohol-related liver disease in asymptomatic individuals. No randomized controlled trials have been conducted to test if screening for liver disease reduces alcohol consumption in individuals with alcohol use disorders, in addition to what can be obtained by motivational interventions. We aimed to assess the feasibility of a randomized controlled trial of a screening for liver disease on the prevalence of alcohol abstinence or light consumption after 6 months in individuals attending outpatient treatment for alcohol use disorder.

**Methods:**

We used an interdisciplinary approach to develop the format of the randomized controlled trial. Individuals were recruited from one outpatient treatment facility for alcohol use disorders. Study participants were randomized 1:1 to receive a) a liver stiffness measurement in addition to usual care (intervention) or b) usual care (control). Follow-up on alcohol consumption was assessed by telephone interview after 6 months and corroborated by data from records from public hospitals and the alcohol treatment facility. Feasibility was assessed by probabilities of recruitment, retention, and completion and estimated by the exact binominal test, with success defined as > 50% participation for each endpoint. The study design was evaluated at interdisciplinary meetings with staff and researchers from the outpatient alcohol treatment facility and the hospital clinic.

**Results:**

Forty of 57 invited individuals agreed to participate in the study (recruitment = 70% (95% CI: 57–82)); 19 of 20 participants randomized to the intervention showed up for the screening (retention = 95% (95% CI: 75–100)). Follow-up telephone interviews succeeded for 33 of 39 reachable participants (completion = 85% (95% CI: 69–94)). Treatment records indicated that the 6 participants who were lost to follow-up for the telephone interview had not achieved alcohol abstinence or light consumption. There was no evidence that the intervention increased abstinence or light alcohol consumption at follow-up: 45% (95% CI: 23–68) in the intervention group and 65% (95% CI: 41–85) in the control group had a alcohol consumption below 10 standard drinks/week at 6 months. The main obstacle regarding study feasibility was to avoid disappointment in individuals randomized as controls.

**Conclusions:**

This feasibility study developed a study design to test the influence of screening for liver disease on abstinence or light alcohol consumption in individuals attending treatment for alcohol use disorder.

**Trial registration:**

ClinicalTrials.gov identifier: NCT05244720; registered on February 17, 2022.

## Key messages regarding feasibility

- What uncertainties existed regarding the feasibility?
  It is unknown whether individuals attending treatment for alcohol use disorder will accept an invitation to a screening for liver disease (study recruitment), whether they will show up for a screening for liver disease at the hospital (study retention) and participate in follow-up 6 months later (study completion).
- What are the key feasibility findings?
  The predefined criteria of feasibility were met according to recruitment, retention, and completion. Those who were lost to follow-up for the telephone interview had evidence of alcohol consumption above the light consumption level in their treatment records. There were reports of disappointment among those randomized as controls, and there were no suggestions of an improved chance of abstinence or light alcohol consumption in those who were randomized to the screening for liver disease.
- What are the implications of the feasibility findings for the design of the main study?
  The study design was considered overall feasible with some modifications. Those randomized as controls will be offered a screening for liver disease by blood sampling. Follow-up data will be collected with information from treatment records from the alcohol facility and hospital, in addition to the telephone interview, to approximate follow-up in 100% of participants. Even if a screening for liver disease does not improve alcohol outcomes, this is important knowledge to avoid unnecessary expenditures on healthcare.

## Background

Alcohol-related liver disease is a common cause of morbidity and mortality among people with alcohol use disorders and causes 500,000 deaths per year globally [1–3]. Today, around 35% of patients with alcohol-related liver disease die within the first year after diagnosis [4]. These deaths might have been avoided if patients were diagnosed earlier. Liver disease usually develops over many years prior to the time of diagnosis and evolves from simple liver steatosis to progressive liver fibrosis and cirrhosis [5]. If patients reduce alcohol consumption before symptomatic liver disease has developed, the liver will often regenerate, and symptomatic liver disease will not develop [5].

A liver stiffness measurement is a key tool to screen for the early stages of alcohol-related liver disease: it is non-invasive and involves no physical risk, it is fast, lasting 5 min, and the result is readily available [6]. International guidelines recommend screening for alcohol-related liver disease in individuals who are hazardous drinkers [6, 7]. Still, screening for liver disease has not been implemented on a larger scale either in Denmark or in other countries [8, 9]. This is due to the lack of evidence regarding the impact of screening on the prognosis in individuals with alcohol use disorders [9]. If a screening procedure for liver disease were to improve the prognosis for patients with unknown alcohol-related liver disease, it should lead to a reduction in alcohol consumption since continuous alcohol consumption is the main driver of disease progression [5]. No randomized controlled trials (RCTs) have been conducted to test if screening for liver disease reduced alcohol consumption in individuals with alcohol use disorders, in addition to what can be obtained by motivational interventions [10]. Therefore, we find it essential to evaluate the impact of screening for liver disease on alcohol consumption in individuals with alcohol use disorder in an RCT. Individuals with prolonged alcohol consumption may have depletion in vitamins and minerals due to an insufficient diet and may need supplements. Therefore, it may be a good idea to draw blood samples and test for micronutrient deficiencies.

The ‘teachable moment hypothesis’ suggests that health problems and perceived consequences of heavy drinking increase the readiness to change drinking behavior in treatment-seeking people with alcohol use disorders [11, 12]. There is also observational evidence that patients lower their alcohol consumption following a diagnosis of asymptomatic liver disease [13]. On this background, our research hypothesis for a future RCT is that screening for liver disease increases the likelihood of abstinence in individuals with hazardous alcohol consumption.

About 20% of individuals attending treatment for alcohol use disorder may have undetected significant liver disease and they are presumably more motivated for behavior change than are for instance patients seen in a gastroenterology department [14, 15]. In order to conduct a full-scale RCT, it is valuable to know whether individuals attending treatment for alcohol use disorder will accept an invitation to a screening for liver disease (study recruitment), whether they will show up for a screening for liver disease at the hospital (study retention) and participate in follow-up 6 months later (study completion).

The objective of this trial was to assess recruitment, retention, and completion in a randomized controlled feasibility trial of a screening intervention for liver disease on alcohol abstinence or light consumption (10 standard drinks/week) after 6 months since randomization in individuals attending outpatient treatment for alcohol use disorder.

## Methods

### Trial design

**Fig. 1:**
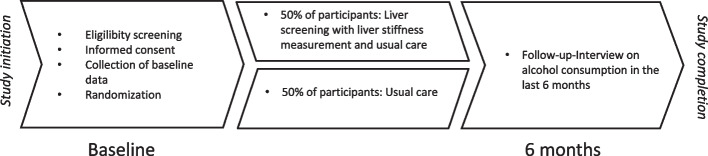
Study design of the feasibility study of “The LIVER CARE trial – screening for liver disease in individuals attending treatment for alcohol use disorder”

This study was a randomized controlled feasibility trial with allocation concealment and blinded outcome assessment of a non-blinded intervention with a two-parallel group design in individuals attending treatment for alcohol use disorder comparing (A) a screening for liver disease with a liver stiffness measurement, physical examination, blood sampling, and an educational leaflet in addition to usual care, vs. (B) usual care (control) (Fig. 1). The reporting of the feasibility trial followed the CONSORT 2010 statement for feasibility studies [16]. No changes to the methods of the feasibility study were implemented after commencement of the study.

### Participants

Study participants were eligible if they attended outpatient treatment for alcohol use disorder. Inclusion criteria were age > 18 years, attending alcohol treatment for less than 6 months, and the ability to give informed written consent. Exclusion criteria were severe liver disease known by the participant, life expectancy of less than 6 months, or inability to speak Danish.

All Danish citizens have access to universal, tax-financed healthcare including outpatient treatment for alcohol use disorder [17]. All participants of the feasibility trial were recruited from a single alcohol outpatient treatment facility, Novavi Køge, with around 200 individuals attending treatment for alcohol use disorder every year. Novavi is a not-for-profit organization that runs open outpatient treatment facilities in the greater capital area of Denmark and has previously participated in several research studies [18, 19]. Novavi offers psychosocial treatment based on motivational interviewing and cognitive behavioral therapy, and if needed, in combination with pharmacological treatment for alcohol use disorder. Individuals seeking treatment for alcohol use disorder can either be referred by their general practitioner or self-refer. All participants in this trial received usual care for alcohol use disorder at the outpatient treatment facility, and those randomized as controls received nothing in addition to this usual care.

Baseline variables included: current alcohol consumption per week in standard drinks (12 g of pure ethanol), date of last alcohol consumption, years with heavy alcohol consumption (defined as years with more than 10 standard drinks/week), sociodemographic variables, and smoking status. In addition, participants were asked about their motivation to change alcohol consumption habits and their belief in this change using a scale from 1 to 10, with higher numbers indicating stronger motivation. The questions regarding motivation are in accordance with the guideline on alcohol use disorders to general practitioners [20].

### Randomization

#### Sequence generation

The randomization sequence was generated by coauthor NW using randomlist.com before enrollment to the trial began. Participants were randomized 1:1 to a) an invitation to screening for liver disease in addition to usual care (intervention), or b) usual care (control).

#### Allocation concealment mechanism

To ensure allocation concealment, a sequentially numbered, sealed opaque envelope with the randomization outcome was provided for each included participant.

#### Implementation

One physician in the alcohol treatment facility (coauthor MKK) screened potential study participants for eligibility, obtained the informed consent, and collected baseline values of participants. Afterwards, MKK handed the opaque envelope with the randomization outcome to the participant.

#### Intervention

Participants randomized to the intervention were contacted by the researcher (coauthor NW) by phone within 1 week after the randomization in order to plan the screening assessment, preferably within 1 month after the randomization. The screening for liver disease was performed at Zealand University Hospital and included a liver stiffness measurement, a physical examination focused on signs of decompensated liver disease (ascites, palmar erythema, etc.), blood tests, and an educational leaflet explaining the physical and mental health benefits of reduced alcohol consumption and abstinence. There was no study manual with instructions for the delivery of the intervention.

Liver stiffness was assessed with the FibroScan© through an intercostal space during a breath hold, with the individual in the supine position and with his/her right arm above the head, following the instructor guideline for the FibroScan©. The liver stiffness measurement was performed by a physician (coauthor NW) who explained the result of the measurement to the participant during the visit. Significant liver fibrosis was defined according to the guideline of the European Association of the Study of Liver Disease as a test result of ≥ 8.0 kPa with 10 successful measurements and an interquartile range of the median < 30% [21]. Blood tests included liver enzymes, blood count, and selected vitamins (folate and cobalamin) and minerals (magnesium and zinc). Individuals with a liver stiffness measurement of ≥ 8.0 kPa were referred to the hepatology clinic for further evaluation.

#### Outcomes

Follow-up after 6 months included all randomized participants and involved (a) one telephone interview, (b) assessment of records from the alcohol treatment facility, and (c) assessment of records from any hospital contact to a public hospital. In the telephone interview, a blinded research nurse obtained the detailed alcohol history for the last 6 months by the validated Timeline Follow-back Method [22]. The research nurse instructed the participant not to reveal his/her treatment allocation at the beginning of the interview. Records from the outpatient alcohol facility were assessed for information on alcohol consumption and untimely treatment drop-out. Medical records were assessed for information on alcohol consumption and evaluations for liver disease in the 6 months after randomization. The outcome, alcohol abstinence or light consumption (10 standard drinks/week) at 6 months, was measured as the average alcohol consumption during the last month before follow-up.

#### Feasibility trial outcomes

The feasibility trial was designed to evaluate and optimize the intervention components before a potential full-scale RCT [23]. The elements to be evaluated in the feasibility study were the recruitment procedure, inclusion/exclusion criteria, questionnaire for collection of baseline variables, the randomization procedure, the recruitment and consent for study participation, the retention of participants in the study, and the follow-up procedure. We assessed the proportion with abstinence or light controlled alcohol consumption at 6 months. Moreover, we wanted to assess cross-over: Would participants randomized to the control group (no evaluation of liver disease) be evaluated for liver disease at the hospital following, e.g., a referral from their general practitioner?

The goals for the feasibility endpoints of recruitment, retention, and completion (Table 1) were set to indicate whether a larger randomized trial would be practically and economically realistic. The final decision to conduct a larger RCT would, however, depend on an overall judgment after a detailed evaluation of the feasibility trial [24, 25].

**Table 1:** Assessment of study feasibility in the feasibility study of “The LIVER CARE trial – screening for liver disease in individuals attending treatment for alcohol use disorder”

	Predefined goal	Achieved in feasibility study
Recruitment — proportion of those invited to participate gives written informed consent	50%	70% (95% CI: 57–82)
Retention — proportion recruited and randomized to the intervention, will attend to the intervention	50%	95% (95% CI: 75–100)
Completion — proportion of all randomized patients to complete the follow-up interview after 6 months	50%	85% (95% CI: 69–94)

### Sample size

We undertook a sample size calculation to ensure that the feasibility study could answer our endpoints regarding feasibility outcomes of recruitment, retention, and completion shown in Table 1 [23, 26]. With the expectation that 75% of those recruited and randomized to the intervention group would show up for a screening for liver disease, we needed to include 40 participants to have 95% confidence limits above the goal for retention of 50% calculated by the exact binominal test: (75% (95% CI: 51–91%)).

### Statistical methods

We conducted descriptive analyses of recruitment, retention and completion with the exact binominal test. Results are reported as percentages with 95% confidence intervals. Analyses were conducted in R.

## Results

### Participant flow

**Fig. 2:**
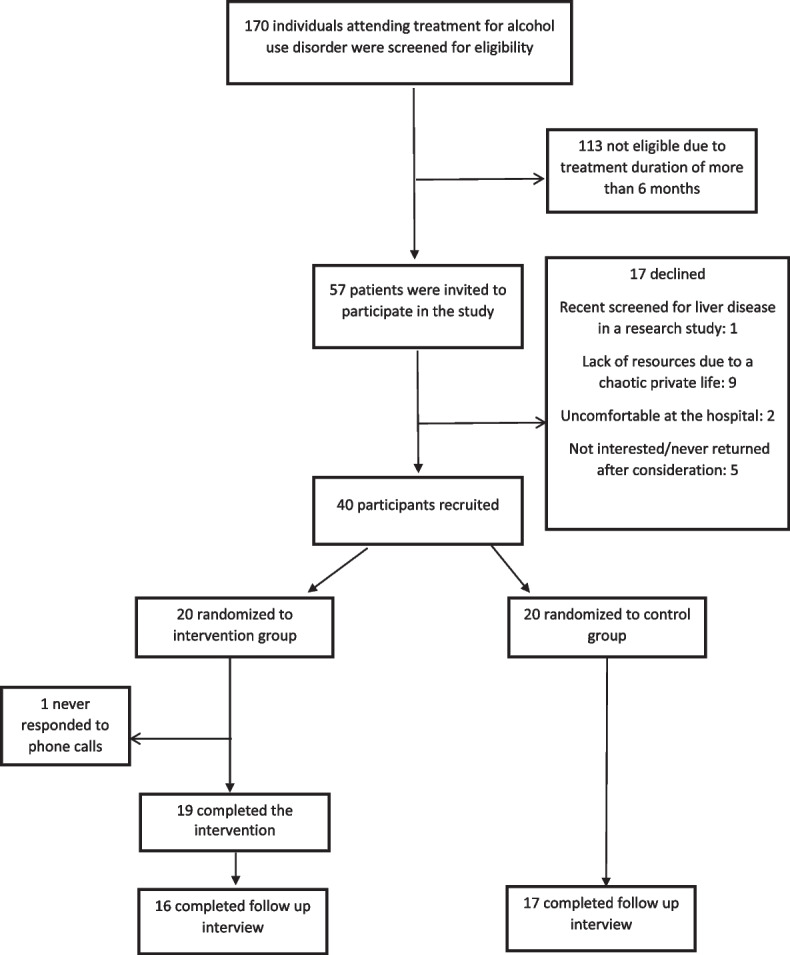
Participant flow of the feasibility study of “The LIVER CARE trial – screening for liver disease in individuals attending treatment for alcohol use disorder”

In total, 170 individuals attended treatment for alcohol use disorder, and 57 of them fulfilled the inclusion criteria and were invited to participate in the feasibility study (Fig. 2). Of these 57 individuals, 17 declined for various reasons with the most common reason being an unstable social situation (*n* = 9). With 40 of the 57 invitees ending up participating, the recruitment was 70% (95% CI: 57–82) (Table 1).

### Recruitment

The recruitment of 40 participants took 4 months from 1 November 2021 until 29 March 2022. Follow-up procedures began on 17 May 2022 and ended on 30 September 2022.

### Baseline data

The majority of those included were men in both the intervention (65% men) and the control group (80% men), and the median age was 46 years in the intervention group and 48 years in the control group (Table 2). The median number of years of heavy alcohol consumption was 10 (range: 1–35) in the intervention group and 13 (range: 0–60) in the control group. Abstinence at baseline was reported in 11 (55%) in the intervention group and 15 (75%) in the control group. Among the participants who were current alcohol consumers, the median consumption was 36 and 43 standard drinks per week in the intervention and control groups. Four of 19 (21%) participants who had a liver stiffness measurement performed had a value indicating significant fibrosis (≥ 8 kPa). Micronutrient deficiencies were detected in 3 out of 17 (18%) participants who had blood samples drawn.

**Table 2:** Baseline data of study participants from the feasibility study of “The LIVER CARE trial – screening for liver disease in individuals attending treatment for alcohol use disorder”, *n* = 40

	Intervention *N* = 20	Controls *N* = 20
Male sex, *n* (%)	13 (65%)	16 (80%)
Age in years, median/mean (range)	46 (24–75)	48 (27–79)
Alcohol abstinent *last week*, *n* (%)	11 (55%)	15 (75%)
Time since last alcohol consumption in days *in current abstainers*, median (range)	27 (0–368)	34 (1–191)
Current alcohol consumption (drinks/week) in *current drinkers*, median (range)	36 (7–140)	43 (1–140)
Years of heavy drinking, median (range)	10 (1–35)	13 (0–60)
Wish to abstain/cut down on scale 1 to 10^a^, median (range)	9.7 (8–10)	9.5 (5–10)
Believe in own abilities to cut down on scale 1 to 10^a^, median (range)	8.5 (5–10)	8.7 (5–10)
Current smoker, *n* (%)	9 (45%)	10 (50%)

^a^Higher values indicate higher wish to abstain/higher believe in ability in to cut down

### Retention

Of 40 included participants, 20 were randomized to the intervention, and 20 were randomized to usual care. One of 20 participants from the intervention group did not present at the hospital for the liver stiffness measurement, giving a retention of 95% (95% CI: 75–100). Of the 19 participants who completed the liver stiffness measurement, 17 had blood samples drawn at the laboratory.

### Completion

Follow-up procedures began on 17 May 2022 and ended on 30 September 2022. After enrollment in the study, 1 participant died, leaving 39 participants eligible for the follow-up interview. Of the 39 participants approached for a telephone interview, this succeeded for 33 out of 39 participants [completion of 85% (95% CI: 69–94)] with a median number of call attempts of 3.

### Abstinence or light alcohol consumption at follow-up

Based on follow-up by telephone interview alone, abstinence or light alcohol consumption was reported in 9 of 16 (56%) participants in the intervention group and in 13 of 17 (76%) participants in the control group (Table 3). These proportions at follow-up were similar to the proportions reporting abstinence at the study baseline (55% and 75%, respectively). Hospital medical records alone did not give evidence of alcohol consumption above the light consumption level in any participant (Table 4). Based on records from the treatment facility for alcohol use disorder, all of the seven participants not approachable for the telephone interview, including the one who have died, had evidence of alcohol consumption above the light consumption level. If data from medical records and the alcohol treatment facility were combined with data from the interview, ensuring follow-up in all 40 participants, abstinence or light alcohol consumption at follow-up was present in 9 of 20 (45% (95% CI: 23–68)) in the intervention group and 13 of 20 (65% (95% CI: 41–85)) in the control group.

**Table 3:** Results from follow-up with interview on alcohol consumption of study participants in the feasibility study of “The LIVER CARE trial – screening for liver disease in individuals attending treatment for alcohol use disorder”, *n* = 40

	Intervention	Controls
Number followed up	16	17
Lost to follow up	4	3
Call attempts^a^, median (range)	3 (1–5)	3 (1–4)
Abstinent/light drinking^b^ last month (yes/no)	9 of 16 (56%)	13 of 17 (76%)
Abstinent/light drinking last month if lost to follow-up is interpreted as alcohol relapse (yes/no)	9 of 20 (45%)	13 of 20 (65%)

^a^Call attempts were on separate days

^b^Light drinking was defined as drinking < 10 standard drinks/week

**Table 4:** Comparison of three follow-up methods to detect abstinence/light drinking^a^ after 6 months for study participants in the feasibility study of “The LIVER CARE trial – screening for liver disease in individuals attending treatment for alcohol use disorder”, *n* = 40

	Number (%) with abstinence/light drinking at 6 months			
	Interview^b^	Hospital medical records^c^	Treatment facility for alcohol use disorder records^d^	Any source indicates abstinence/light drinking
Interview	22 of 33 (67%)	33 of 33 (100%)	21 of 33 (64%)	21 of 33 (64%)
Lost to follow-up for interview	-	7 of 7 (100%)	0 of 7 (0%)	0 of 7 (0%)
All	-	40 of 40 (100%)	21 of 40 (53%)	21 of 40 (53%)

^a^Abstinence/light drinking (< 10 standard drinks/week) was assessed as the average last month

^b^Telephone interview with the study participant was conducted by a research nurse with the timeline follow-back method

^c^Hospital medical records from all public hospitals were assessed for information on alcohol use

^d^Records from the treatment facility where the study participant had been recruited were assessed for information on alcohol use and untimely treatment drop-out

### Time commitment and logistics

It took around 20 min per included participant for the recruiting physician to screen, inform, obtain written consent, collect baseline variables, and deliver the envelope with the randomization outcome. The hospital visits with the screening for liver disease, physical examination, liver stiffness measurement, oral and written information to the study participant about the interpretation of the screening of liver disease, and drawing of blood samples took on average 30 min. The telephone follow-up interview performed by an experienced research nurse took 20 min on average.

### Overall facilitators and barriers to protocol implementation

Several participants who were randomized as controls expressed disappointment with not receiving screening for liver disease. However, no cross-over from the control to the intervention group was observed, i.e., none of those randomized as controls have had a transient elastography or other kind of assessment for liver disease at a Danish hospital when assessed by electronic medical chart review in November 2022.

### Harms

No harms to participants were recorded.

## Discussion

This randomized controlled feasibility trial of a screening for liver disease in individuals attending outpatient treatment for alcohol use disorder showed that the predefined criteria of feasibility were met according to recruitment, retention, and completion. However, the feasibility trial pointed to potential problems related to the conditions for those randomized as controls. There was no tendency to an effect of the screening for liver disease on abstinence or light controlled alcohol consumption at the 6-month follow-up.

This feasibility trial showed a recruitment of 70%, comparable with the recruitment of 60–88% from studies undertaken in treatment-seeking individuals with alcohol use disorder in Denmark [19, 27, 28]. The study population had a similar distribution of sex and age as reported for a large sample of treatment-seeking individuals in Novavi outpatient alcohol treatment facilities [29]. We are aware that the recruitment could be lower when undertaken by several healthcare professionals in the main study instead of one motivated physician. We will seek to increase recruitment in the main study by face-to-face meetings and teaching sessions with the recruiting staff [30, 31]. Also, we will remove the inclusion criterion of less than 6 months of treatment for alcohol use disorder, as this was the main obstacle to fulfilling the study inclusion criteria (Fig. 2). The less strict inclusion criteria and the recruitment by several healthcare professionals will add external validity to the study [32]. The high recruitment into the study may be due to the simple study design, the close collaboration between researchers and referral staff, and importantly, the fact that time resources related to screening and recruitment were reimbursed equal to 1 h per recruited participant to the outpatient alcohol treatment facility [30, 31].

With regard to follow-up, our result of 85% having the follow-up interview is comparable with the 50–76% reported in studies with similar study populations [19, 28], but should rather approximate 100% to minimize the influence of attrition bias [33]. Indeed, all those seven study participants lost to follow-up for the telephone interview had evidence of alcohol relapse in their records from the alcohol treatment facility (Table 4), highlighting the importance of getting follow-up data for all participants in the study.

Some participants of the feasibility trial were disappointed with randomization to usual care, i.e., not being offered a screening for liver disease. Disappointment in the control group could introduce bias, such as selective withdrawal of controls, poor response to follow-up interviews, and cross-over with controls pursuing a screening for liver disease through their general practitioner [34]. To combat disappointment in the control group, controls will be offered a screening for liver disease by blood tests in the main study, as is recommended in the European guideline for non-invasive testing of alcohol-related liver disease [6]. The controls will need to arrange the blood tests themselves and will not have any face-to-face contact with a physician unless a blood test result is abnormal; this procedure will mimic the usual contact with a general practitioner. We are aware that this control group condition may cause us to underestimate the effectiveness of the active intervention compared to what can be expected in real-world practice [35].

The feasibility trial did not show any tendency towards a higher prevalence of abstinence or light alcohol consumption in the intervention compared to the control group at follow-up. The overall proportion of 22 of 40 (55%) with this favorable outcome is similar to what has been reported in a European study, also recruiting participants from Denmark, in which abstinence or light alcohol consumption was found in 51% (268 of 530 study participants) at follow-up [28]. The lack of association in our study between screening for liver disease and abstinence or light controlled consumption at follow-up could also be a chance finding due to the low number of study participants. In the larger RCT, we will investigate if there is a reduction in overall alcohol consumption, however, this will be restricted to those who have the interview in contrast to the main outcome of abstinence or light controlled consumption that can be evaluated in all participants, regardless of the follow-up interview. It also led us to consider if the effect of the intervention could be enhanced if the FibroScan was delivered in the spirit of motivational interviewing, so we decided to write a manual based on motivational interviewing for the larger RCT. However, even if a screening for liver disease does not improve alcohol outcomes, this is important knowledge. There is a strong interest from the European research community to implement screening for liver disease among individuals with a harmful alcohol consumption [6, 8, 9]. According to good clinical practice, screening for liver disease should only be implemented if the benefits balance the harms and costs related to the screening [36]. For example, it speaks against an implementation of screening if evidence suggests that screening has no effect on the patient’s prognosis. Finally, 18% of the participants had micronutrient deficiencies. Research should address if individuals in alcohol misuse treatment benefit from a general recommendation of a standard multivitamin tablet or if blood tests to test for specific deficiencies are a better approach.

In conclusion, this study showed overall feasibility according to recruitment, retention, and completion of a larger RCT investigating the impact of screening for liver disease on abstinence or light consumption in individuals attending treatment for alcohol use disorder. Another implication is to study whether screening for liver disease will lead to a reduced liver-related death in the screened individuals. We expect the proposed randomized controlled study to cause minimal harm to the participants. The study will answer an important research question about an overall net benefit of screening for liver disease in individuals attending treatment for alcohol use disorders. Therefore, the overall benefits of conducting this study are much greater than the expected harms.

## Data Availability

The datasets analyzed during the current study are not publicly available due to the small number of participants and data protection.
